# Supplementary material for: Longitudinal Association between Sarcopenia and Cognitive Impairment among Older Adults in Rural Malaysia
Source: Int J Environ Res Public Health. 2022 Apr 14;19(8):4723. doi: 10.3390/ijerph19084723 (PMC9025848; doi:10.3390/ijerph19084723)
Supplement: Supplementary file 1 [file ijerph-19-04723-s001.zip › ijerph-1625001-supplementary.pdf]

Supplementary table

Table S1 Longitudinal association of sarcopenia and cognitive impairment

Exposure	Unadjusted model			Fully adjusted model[3]		
	b	Crude RR[2] (95% CI)	P value	b	Adjusted RR[2] (95% CI)	P value
Sarcopenia						
No		1			1	
Yes	0.76	2.13(1.46-3.09)	<0.001	0.59	1.80(1.18-2.75)	0.01
Age						
60-69		1			1	
70-79	0.19	1.21(0.95-1.53)	0.12	0.10	1.10(0.86-1.41)	0.45
≥80	0.71	2.04(1.5-2.78)	<0.001	0.27	1.3(1.1-1.88)	0.02
Gender						
Male		1			1	
Female	0.18	1.19(0.96-1.48)	0.11	0.15	1.16(0.92-1.46)	0.22
PASE Quartiles1						
Q4		1			1	
Q3	0.06	1.06(0.72-1.57)	0.77	0.01	1.01(0.68-1.5)	0.97
Q2	0.44	1.56(1.07-2.27)	0.02	0.37	1.44(0.98-2.12)	0.06
Q1	0.88	2.42(1.74-3.36)	<0.001	0.63	1.87(1.32-2.66)	<0.001
Depressive symptoms		1			1	
No	0.77	2.15(1.68-2.75)	<0.001	0.74	2.10(1.63-2.7)	<0.001
Yes						
Chronic pain						
No		1			1	
Yes	0.54	1.72(1.35-2.19)	<0.001	0.33	1.39(1.08-1.79)	0.01
Visual Impairment						
No		1			1	
Yes	0.20	1.22(0.97-1.53)	0.09	0.02	1.02(0.8-1.3)	0.86
Hearing Impairment		1			1	
No	0.53	1.69(1.25-2.28)	<0.001	0.31	1.37(1.05-1.91)	0.04
Yes						

[1] Q: Quartile

[2] RR : Relative Risk

[3]Fully adjusted model, adjusted for age, gender, physical activities, depressive symptoms, chronic pain visual and hearing impairment.

Table S2 Longitudinal association of severe sarcopenia and cognitive impairment

Exposure	Unadjusted model			Fully adjusted model[3]		
	b	Crude RR[2] (95% CI)	P value	b	Adjusted RR[2] (95% CI)	P value
Severe Sarcopenia						
No		1			1	
Yes	1.05	2.85(1.95-4.15)	<0.001	0.77	2.01(1.24-3.27)	<0.001
Age						
60-69		1			1	
70-79	0.18	1.2(0.95-1.5)	0.12	0.12	1.12(0.88-1.43)	0.34
≥80	0.66	1.94(1.43-2.63)	<0.001	0.29	1.34(0.92-1.96)	0.13
Gender						
Male		1			1	
Female	0.23	1.26(1.02-1.55)	<0.001	0.31	1.36(1.05-1.76)	0.02
PASE Quartiles1						
Q4		1			1	
Q3	0.01	1.01(0.69-1.48)	0.96	-0.08	0.92(0.63-1.36)	0.69
Q2	0.42	1.52(1.06-2.18)	0.02	0.32	1.38(0.95-1.99)	0.09
Q1	0.86	2.37(1.72-3.25)	<0.001	0.74	2.1(1.5-2.94)	<0.001
Depressive						
symptoms		1			1	
No	0.77	2.15(1.68-2.75)	<0.001	0.73	2.09(1.62-2.68)	<0.001
Yes						
Chronic pain						
No		1			1	
Yes	0.54	1.72(1.35-2.19)	<0.001	0.33	1.39(1.08-1.79)	0.01
Visual Impairment						
No		1			1	
Yes	0.19	1.21(0.97-1.51)	0.09	0.10	1.11(0.88-1.4)	0.38
Hearing						
Impairment		1			1	
No	0.51	1.66(1.24-2.22)	<0.001	0.37	1.45(1.04-2)	0.03
Yes						

[1] Q: Quartile

[2] RR : Relative Risk

[3]Fully adjusted model, adjusted for age, gender, physical activities, depressive symptoms, chronic pain visual and hearing impairment.
